# Comparative evaluation of immediate versus delayed loading in dental implants

**DOI:** 10.6026/973206300220583

**Published:** 2026-01-31

**Authors:** Alisha Alam, Satish Makwana, Arun Kharavela Mohanty, Hina Kausher, Vinod Venugopal Menon, Gatha Mohanty

**Affiliations:** 1 Department of Oral Maxillofacial Surgery, Jaipur Dental College, Rajasthan, India; 2 Department of Dentistry, CU Shah Medical College and Hospital, Surendranagar, Gujarat, India; 3 Department of Prosthodontics, Kalinga Institute of Dental Sciences, KIIT University, Bhubaneswar, Odisha, India; 4 Department of Applied Medical Sciences, College of Pharmacy, Nursing and Medical Sciences, Riyadh Elm University, Riyadh- Kingdom of Saudi Arabia; 5 Department of Prosthodontics, New Look Medical Centre, Al Ain, UAE; 6 Department of Periodontics and Oral Implantology, Institute of Dental Sciences, Shiksha 'O' Anusandhaan University, Bhubaneswar, Odisha, India

**Keywords:** Immediate loading, delayed loading, dental implants, implant survival rate, marginal bone loss

## Abstract

The optimal timing of prosthetic loading for dental implants remains a clinical challenge, with ongoing debate regarding the long-term outcomes of immediate versus delayed loading protocols. Therefore, it is of interest to compare peri-implant outcomes, marginal bone level changes, and clinical survival rates between immediately loaded and delayed loaded dental implants over a 24-month period. Fifty implants placed in 60 patients were allocated into two groups: immediate loading (n = 25), restored within 48 hours, and delayed loading (n = 25), restored after 8-16 weeks following standardized surgical protocols. Implant survival, implant stability quotient (ISQ), peri-implant clinical parameters, marginal bone levels, complications, and patient-reported satisfaction were evaluated. While immediate loading demonstrated higher early patient satisfaction, both loading protocols showed comparable long-term clinical and radiographic outcomes, indicating that each approach is reliable when appropriate case selection is followed.

## Background:

The success of dental implants, now regarded as a reliable treatment option for replacing missing teeth, depends on several factors, including loading protocols and timing of implant placement. Both immediate and delayed loading strategies have their respective advantages and limitations, leading to ongoing debate in clinical practice. Using cone-beam computed tomography (CBCT), Muthaiyan *et al.* compared immediate and delayed implant placement and reported clinically comparable peri-implant bone levels [1]. A six-year retrospective study further evaluated the influence of patient- and implant- related factors on survival rates and concluded that, with proper case selection, both immediate and delayed approaches can achieve high long-term success [2]. Meta-analyses also strengthen the evidence supporting these findings. One systematic review reported no statistically significant difference in implant survival between immediate and delayed loading, although variations in marginal bone loss were noted across studies [3]. Another review confirmed similar survival rates for both protocols while emphasising the role of implant design and surgical technique in overall treatment outcomes [4]. Clinical observations further align with this evidence. A study evaluating mandibular posterior implants found no notable difference in the clinical performance of immediate versus delayed loading [5]. Likewise, a literature review highlighted that both protocols demonstrate consistently high success, but stressed that treatment planning should consider patient-specific factors such as occlusal forces and bone quality [6]. Collectively, these studies provide a strong foundation for comparing immediate and delayed loading protocols with respect to clinical survival rates and peri-implant outcomes. Therefore, it is of interest to evaluate immediate vs delayed loading in dental implants.

## Methodology:

With a 24-month follow-up, this prospective, parallel-group clinical study was carried out in a university dental hospital's Department of Prosthodontics and Periodontology. The institutional review board granted ethical approval and each participant signed an informed consent form. Fifty implants were inserted into 60 adult patients (≥18 years old) who needed single or short-span implant- supported fixed prostheses. Eligible patients had either non-infected extraction sockets that could be used for immediate implant placement or healed edentulous sites (≥12 weeks), with enough bone volume for implants that were at least 3.5 mm in diameter and 8 mm in length. Patients who were pregnant or nursing, had parafunctional habits without protective splints, untreated periodontitis, heavy smoking (>10 cigarettes per day), uncontrolled systemic disease, or local infections were not included. Each of the two groups of participants received 25 implants. While Group B (Delayed Loading, DL) received healing abutments and was restored after 8-16 weeks, Group A (Immediate Loading, IL) received a provisional or definitive restoration within 48 hours of placement. Allocation balanced factors like smoking status, site and jaw location. After the usual prophylactics and chlorhexidine rinse, all surgeries were carried out under local anaesthesia. Bone quality was recorded and osteotomies were prepared in accordance with the manufacturer's instructions. Implant stability quotient (ISQ) ≥65 or insertion torque ≥35 Ncm were considered indicators of primary stability. When necessary, guided bone regeneration was carried out. While DL patients received healing abutments until definitive prostheses were delivered, IL group patients received screw-retained provisionals that were kept out of occlusion. Nightguards were supplied to bruxers and the final restorations for both groups were either metal-ceramic or CAD/CAM zirconia prostheses that were torqued in accordance with manufacturer recommendations. Analgesics were part of the postoperative care and when guided bone regeneration was done, antibiotics were prescribed. Every six months, all patients were scheduled for supportive professional maintenance and chlorhexidine mouthwashes were advised. Implant survival, which is defined as the lack of pain, mobility, suppuration, or progressive bone loss, was the main outcome at 12 and 24 months. Secondary outcomes included peri-implant clinical indices like plaque index, bleeding on probing, probing depth, keratinised mucosa and Pink Aesthetic Score; peri-implant complications like peri-implant mucositis, peri-implantitis and technical issues; patient- reported outcomes like pain (VAS), aesthetic satisfaction and oral health-related quality of life (OHIP-14); and marginal bone level changes measured on standardised periapical radiographs. Two calibrated examiners (κ/ICC ≥0.80) collected all of the data. Kaplan-Meier survival estimates and log-rank tests for implant survival were used in the statistical analysis. PROMs were examined using independent t-tests or Mann-Whitney U tests, peri-implant clinical outcomes using chi-square, Fisher's exact, or mixed models and changes in marginal bone level using linear mixed-effects models. Using Benjamini-Hochberg correction for secondary outcomes, the significance threshold was set at α = 0.05.

## Results:

Twenty-five implants were placed in the immediate loading group and twenty-five in the delayed loading group, for a total of fifty implants in sixty patients. The overall implant survival rate was 96.0% at the conclusion of the 24-month follow-up period. The two groups did not differ in any way that was statistically significant. Two implant failures in the immediate loading group and one in the delayed loading group were noted overall. The fact that every failure happened within the first six months of implantation highlights how important the early healing phase is to the stability and success of implants over the long run (Table 1). Peri-implant stability was assessed by analysing changes in marginal bone level (MBL). The immediate loading group experienced an average bone loss of 0.82 ± 0.29 mm, while the delayed loading group experienced an average bone loss of 0.79 ± 0.32 mm. The timing of implant loading had no effect on peri-implant bone remodelling during the observation period, according to comparative analysis, which found no statistically significant intergroup difference (p > 0.05) (Table 2). Throughout the two-year follow-up, both groups showed stable marginal bone levels, which is consistent with well-preserved peri-implant conditions. At every observation point, the two protocols produced similar outcomes for peri-implant clinical parameters such as plaque index, bleeding on probing and probing depth. These results showed that regardless of the loading strategy, healthy peri-implant tissues were preserved. As a result of consistent osseointegration and implant integration, the implant stability quotient (ISQ) values in both groups showed progressive increases over time. The comparable biological performance of the immediate and delayed loading protocols was further supported by the similar trends in ISQ values between the two groups. The clinical results were supplemented by patient-reported outcome measures (PROMs). Since this method shortened the duration of edentulism, participants in the immediate loading group expressed higher levels of satisfaction during the early healing phase, particularly with regard to function and aesthetics. However, these differences gradually decreased and by the time the definitive prosthesis was delivered, patient satisfaction scores were similar between the two groups. At the 24-month follow-up, no long-term statistical difference was observed. There were few issues and they were split equally between the two protocols. Peri-implant mucositis was the most common biological complication, but it was successfully treated with expert care and reinforcement of oral hygiene. Crucially, no peri-implantitis cases were noted throughout the investigation. In conclusion, the results of both immediate and delayed loading protocols were positive and consistent. Over a two-year follow-up period, the timing of loading had no discernible effects on patient satisfaction, peri-implant health, marginal bone stability, or implant survival (Table 1). This suggests that both strategies are clinically sound treatment options.

## Discussion:

The findings of this study align with the expanding body of evidence demonstrating that, with appropriate planning, both immediate and delayed implant placement and loading can yield predictable and successful outcomes. Although immediate protocols were once approached cautiously, advancements in implant design, surface technology and digital planning have transformed immediate loading from a selective technique into a reliable option in routine clinical practice. A retrospective multicenter analysis evaluating novel tissue- level implants found comparable survival rates between immediate and delayed placement, highlighting the importance of implant geometry and macro-design in achieving stability across different clinical timings [7]. These results reinforce how technological improvements have mitigated risks traditionally associated with immediate loading. Systematic reviews further support this shift by synthesising evidence from multiple randomised controlled trials. A meta-analysis comparing immediate, early and conventional loading concluded that immediate loading is a dependable modality when cases are properly selected and primary stability is achieved [8]. Another review focusing on post-extraction loading similarly reported high survival rates, emphasising the need to evaluate bone volume, occlusal forces and patient-specific factors for long-term success [9]. Additional prospective and retrospective studies corroborate these observations. A large retrospective study underscored the role of individualised treatment planning by showing that local parameters such as implant stability and keratinised tissue significantly influence peri-implant survival [10]. Likewise, a comparison of healing durations found no meaningful differences in outcomes between immediate and delayed loading, indicating that immediate protocols can be safely implemented under optimal conditions [11]. Prospective clinical research further supports this, with one study reporting favourable functional and aesthetic outcomes for immediate anterior implants when primary stability was ensured [12], while another demonstrated comparable clinical and radiographic results between immediate and delayed approaches [13]. Even in complex clinical scenarios, immediate loading has shown promising results. For example, a systematic review on implant therapy in irradiated patients found that both immediate and delayed loading can be successful with vigilant monitoring and maintenance [14]. Similarly, a meta-analysis evaluating overdenture-supported implants found no significant differences in marginal bone loss between the two protocols, suggesting broad applicability across fixed and removable prosthetic solutions [15]. Long-term prospective follow-ups in posterior regions have also confirmed the stability of immediate implants over 3-5 years, extending confidence beyond aesthetic zones [16]. Immediate loading of dental implants shows survival rates comparable to delayed loading protocols when implants are placed under controlled clinical conditions. The review also reported no clinically significant differences in marginal bone level changes between immediate and delayed loading approaches, supporting the predictability of both protocol [17]. Furthermore, a randomised controlled trial comparing early versus conventional loading validated the safety of accelerated protocols, finding no significant differences in clinical or radiographic parameters [18]. Consistent with these conclusions, a systematic review comparing immediate post- extraction and delayed loading reported no significant differences in survival rates or marginal bone loss [19]. Overall, both immediate and delayed implant loading represent viable and clinically successful strategies. The choice between them should be guided by individual patient factors such as bone quality and volume, implant stability, aesthetic needs, systemic considerations and overall risk profile. While immediate loading offers advantages including reduced treatment time and increased patient satisfaction, delayed protocols remain the preferred option in less ideal conditions. Ultimately, balancing clinical predictability with patient- centred care is essential for achieving long-term functional and aesthetic success.

## Conclusion:

Both immediate and delayed loading protocols demonstrated stable marginal bone levels and high survival rates after 24 months. Without sacrificing long-term results, immediate loading increased patient satisfaction in the short term. Individual clinical factors, such as implant stability, bone quality and aesthetic requirements, should guide the choice of treatment.

## Figures and Tables

**Table 1:** Implant survival data

**Group**	**Number of Implants**	**Failures**	**Survival Rate (%)**
Immediate Loading	25	2	92
Delayed Loading	25	1	96

**Table 2:** Marginal bone loss data

**Group**	**Mean Bone Loss (mm)**	**SD (±)**
Immediate Loading	0.82	0.29
Delayed Loading	0.79	0.32
